# Significantly Delayed Development of Polyarthritis with Active Tenosynovitis after Possible Temporary Neutropenic Immune-Related Adverse Events Caused by Atezolizumab Treatment: A Novel Case Report

**DOI:** 10.1155/2024/1566299

**Published:** 2024-02-08

**Authors:** Yoshitaka Saito, Yoh Takekuma, Hajime Asahina, Ryo Hisada, Mitsuru Sugawara

**Affiliations:** ^1^Department of Clinical Pharmaceutics & Therapeutics, Faculty of Pharmaceutical Sciences, Hokkaido University of Science, 4-1, Maeda 7-jo 15-chome, Teine-ku, Sapporo 006-8585, Japan; ^2^Department of Pharmacy, Hokkaido University Hospital, Kita 14-jo, Nishi 5-chome, Kita-ku, Sapporo 060-8648, Japan; ^3^Department of Respiratory Medicine, Faculty of Medicine, Hokkaido University, Kita 15-jo, Nishi 7-chome, Kita-ku, Sapporo 060-8638, Japan; ^4^Department of Rheumatology, Endocrinology and Nephrology, Faculty of Medicine and Graduate School of Medicine, Hokkaido University, Kita 15-jo, Nishi 7-chome, Kita-ku, Sapporo 060-8638, Japan; ^5^Laboratory of Pharmacokinetics, Faculty of Pharmaceutical Sciences, Hokkaido University, Kita 12-jo, Nishi 6-chome, Kita-ku, Sapporo 060-0812, Japan

## Abstract

Immune checkpoint inhibitors have drastically improved cancer treatment. However, they may induce immune-related adverse events (irAEs). Here, we report a case of significantly delayed rheumatic irAEs (Rh-irAEs) with prior possible temporary neutropenic irAEs in a patient with atezolizumab-treated non-small-cell lung cancer and its management. A man in his sixties received atezolizumab monotherapy as the sixth-line treatment. He experienced an infusion-related reaction (fever) during the first cycle. On day 22 of cycle 2, grade 4 neutropenia suddenly appeared, but it disappeared on the next day. Cycle 3 was initiated after seven days; the patient did not exhibit any symptoms for approximately 500 days. However, on day 534 (day 1 of cycle 21), the patient complained of pain in the shoulders, back, and wrists. On day 644, the shoulder and back pain worsened with obvious swelling of the fingers. We thus suspended treatment and consulted a rheumatologist. A diagnosis of polyarthritis with active tenosynovitis was made based on joint ultrasound and laboratory tests. Prednisolone 15 mg attenuated the symptoms, allowing suspension of analgesics; however, dose reduction from 15 mg/day was difficult because of symptom flares. Finally, iguratimod 25 mg twice daily was initiated on day 764; prednisolone was reduced to 10 mg without flares, and its dosage was slowly reduced to 5 mg/day. Although irAEs exhibit multisystem features, delayed development of polyarthritis with active tenosynovitis after possible temporary neutropenic irAEs is rare. Thus, irAEs need to be monitored for a long time in patients with suspected irAE development even if it appears transiently.

## 1. Introduction

Immune checkpoint inhibitors (ICIs) have drastically improved treatment of various malignancies [1]. However, ICI administration can induce immune-related adverse events (irAEs), which are significantly different from those induced by cytotoxic treatments, in 15–90% of the administered patients [1]. The mechanism of irAEs remains unclear; however, earlier-onset irAEs, such as rash, colitis, and pneumonitis, appear to involve generalized epithelial inflammation, while later-onset irAEs, which are less common, tend to be more localized, organ-specific reactions [1].

Among irAEs, rheumatic symptoms are rather common, with an incidence rate of approximately 5% [2, 3]. The median onset time of rheumatoid irAEs (Rh-irAEs) is reportedly 3–7 months from ICI treatment initiation [2, 4, 5]. A previous report from the Mayo Clinic suggested that their incidence was confirmed in 3.3% of ICI-treated patients (43 of 1293), all of whom developed symptoms within 40 weeks of treatment initiation [2]. Further, Mitchell et al. reported a case series of Rh-irAEs in 36 patients, suggesting that three patients showed significantly delayed symptom onset (after 80 weeks) along with other irAEs such as pancreatitis, hypophysitis, and thyrotoxicosis during the ICI treatment [5].

Here, we report a case of a significantly delayed Rh-irAE after possible temporary neutropenic irAE in a patient with atezolizumab-treated non-small-cell lung cancer and its management.

## 2. Case Report/Case Presentation

A man in his sixties with controlled primary hypertension, type 2 diabetes mellitus, benign prostatic hyperplasia, and reflux esophagitis was diagnosed with stage 4 lung adenocarcinoma (clinical stage T4N2M1a, epidermal growth factor receptor (EGFR) exon 19 deletion, and PDL1 expression of 0%). The patient was initially treated with afatinib for approximately 8 months, which was discontinued because of pneumonia. The second-line treatment was cisplatin + pemetrexed for four cycles, followed by eight cycles of pemetrexed monotherapy. After one cycle of docetaxel + ramucirumab as the third-line therapy, afatinib was administered again for five months. The fifth-line treatment comprised carboplatin + nanoparticle albumin-bound paclitaxel (nab-PTX) for three cycles. After disease progression on the fifth-line treatment, palliative radiation therapy was performed for right rib bone metastasis (25 Gy/5 fractions). Sixth-line atezolizumab monotherapy was initiated 104 days after the last nab-PTX administration and 12 days after the last radiation dose.

The baseline medication was teneligliptin 20 mg once a day, esomeprazole 20 mg per day, trichlormethiazide 1 mg and valsartan 40 mg once daily, rosuvastatin 2.5 mg once a day, tamsulosin hydrochloride 0.2 mg once a day, eszopiclone 2 mg at bedtime, mirogabalin besilate 7.5 mg twice a day, magnesium oxide 330 mg twice to three times a day, dihydrocodeine phosphate 10 mg three times a day, clostridium butyricum 40 mg three times a day, and acetaminophen 500 mg as needed for managing thoracic pain. These medications, except for mirogabalin, were continued at the same dose for six months before atezolizumab induction.

His baseline subjective symptoms were grade 1–2 peripheral neuropathy at the bottom of his foot, grade 1 coughing, and thoracic pain with coughing, all of which were well managed with medication.

On day 2 and for several days from day 8 in the first cycle of atezolizumab treatment, the patient experienced fever without any change in laboratory data; however, these symptoms did not appear after the second cycle of treatment. Therefore, we concluded that this was an infusion-related reaction. On day 22 of cycle 2 (day 43 from treatment initiation), grade 4 neutropenia suddenly appeared (without any neutropenic variation until the day) and the treatment was suspended, but it disappeared on the next day after one subcutaneous injection of lenograstim 100 *μ*g. Cycle 3 was resumed seven days later, and the symptoms did not reappear. Apixaban 10 mg twice a day for seven days followed by 5 mg twice daily was initiated for treating deep vein thrombosis in the lower extremity on day 1 of the third cycle. On day 1 of cycle 4, valsartan was discontinued because of a decrease in blood pressure, and mirogabalin was reduced to 5 mg twice daily, resulting in blood pressure elevation without neuropathy degradation. Additionally, denosumab 120 mg subcutaneous injection was initiated from day 1 of the seventh cycle (day 148) for treating bone metastasis. Oral calcium/vitamin D supplementation (610 mg calcium and 400 IU cholecalciferol per day) was coadministered from the same day to prevent hypocalcemia. Atezolizumab monotherapy and other medications were regularly administered at the same dose for approximately one year after denosumab initiation without any adverse effects.

However, on day 534 (day 1 of cycle 21), the patient complained of discomfort and pain in both shoulders, back, and right wrist. He received as-needed acetaminophen (500 mg), and treatment was carefully continued. The symptoms were attenuated by acetaminophen 2–3 times per day, and the 22nd and 23rd cycles were administered with careful monitoring. After twenty-one days, the symptoms worsened, and the patient experienced mild difficulty turning over in bed because of shoulder pain. He also complained of pain in both wrists and moderate morning stiffness in the fingers. Loxoprofen (60 mg) was administered twice daily, and the 24th treatment session was cautiously performed. However, the pain in the shoulders and back worsened, and obvious finger swelling appeared after three weeks (day 644). The 25th cycle was thus suspended, and a rheumatologist was consulted.

Joint ultrasound was performed, and active synovitis was observed in both fingers, wrists, acromioclavicular joints, and the left sternoclavicular joint. In addition, tenosynovitis was observed in the right long head of the biceps brachii and extensor compartment (4 for both, 2 and 6 for the left). Finally, the patient was diagnosed with grade 2 polyarthritis with active tenosynovitis on day 645. Serum laboratory data on day 645 showed abnormal values for IgA (453 mg/dL), IgG (2510 mg/dL), IgM (186 mg/dL), complement C3 (180 mg/dL), complement C4 (46 mg/dL), 50% hemolytic complement activity (90.7 U/mL), anti-nuclear antibody (40-fold), SS-A antibody (over 2200 U/mL), PR3-ANCA (15.0 U/mL), MMP-3 (430.5 ng/mL), and C-reactive protein (3.08 mg/dL) (Table 1). In contrast, the levels of MPO-ANCA (<0.3 U/mL), rheumatoid factor (<4.5 IU/mL), uric acid (5.7 mg/dL), and anti-citrullinated peptide antibody (negative) were in the normal range. The patient did not exhibit dryness of the eyes or mouth despite high levels of SS-A antibody, suggesting that Sjögren's syndrome involvement is less likely. Prednisolone 15 mg once daily with trimethoprim 80 mg + sulfamethoxazole 400 mg was initiated on the day of diagnosis (day 645). The range of joint motion increased with improvement in pain at the shoulders, back, and wrist; swelling and pain at the finger joints were also attenuated from the next day, allowing suspension of loxoprofen and acetaminophen after several days. After 29 days of prednisolone initiation (day 674), the dosage was reduced to 12.5 mg a day and to 10 mg/day after 14 days. However, after dose reduction to 10 mg, the symptoms flared to the baseline levels without pain relief with loxoprofen and acetaminophen. The prednisolone dosage was thus increased to 15 mg/day 20 days after the reduction (day 708), with significant symptom relief within several days. We attempted to reduce the prednisolone dosage to 12.5 mg again after 4 weeks of administering 15 mg (day 736); however, this resulted in a mild flare. Finally, iguratimod 25 mg twice daily was initiated on day 764, and prednisolone was reduced to 10 mg without flares and slowly reduced to 5 mg/day. The patient did not exhibit any symptoms in the lower extremities. Atezolizumab treatment was not resumed because of Rh-irAEs, and disease progression at supraclavicular lymph nodes and the right adreno was confirmed on day 764.

## 3. Discussion

Although irAEs are mild and manageable in most cases, they can rarely become severe or life threatening. The characteristic features of irAEs are diversity, multifocality, and continuity [6, 7]. Further, irAE development is associated with the better efficacy of ICI therapy [3–5]; therefore, appropriate irAE diagnosis and management with sufficient comprehension are important for improved treatment.

The articular symptoms in this case were assumed to be irAEs. Other differentials included a paraneoplastic etiology, reactive and other seronegative arthritis, and pseudogout; however, the timing of symptoms was in keeping with an irAE. Rh-irAEs are uncommon symptoms, reportedly appearing in <10% of ICI-administered patients [2, 3]; however, we have rarely observed these symptoms. Inflammatory arthritis, inflammatory myopathy, sicca syndrome, polymyalgia- (PMR-) like syndrome, and tenosynovitis are representative Rh-irAEs [2, 4, 8]; however, the incidence of tenosynovitis has not been circumstantially reported. The reported median onset time of Rh-irAEs is approximately 3–7 months after treatment initiation, suggesting that they appear relatively later in the treatment phase [2, 4, 5]. In addition, Richter et al. reported that approximately 70% of patients with Rh-irAEs experience other irAEs, such as rash, colitis, neuritis, hepatitis, pneumonitis, thyroiditis, and hypophysitis [2]. In a previous study, all patients exhibiting significantly delayed Rh-irAEs (80 weeks after initiation) during ICI treatment developed other irAEs such as pancreatitis, hypophysitis, and thyrotoxicosis prior to the symptoms [5]. Furthermore, Rh-irAE-exhibiting patients achieve longer cancer treatment benefits from ICIs [3–5]. Consequently, we speculate that almost all patients with significantly delayed Rh-irAEs have exhibited other prior irAEs. In this case, the patient developed an infusion-related reaction, and temporary neutropenic symptoms were suspected. We considered that the infusion-related reactions were different from irAEs; we hypothesized that the temporary neutropenic symptoms were hematological irAEs considering the treatment process and reported them even though a definite diagnosis could not be made owing to rapid symptom resolution [9]. The patient exhibited polyarthritis with tenosynovitis after submission of the previous manuscript, supporting our speculation that temporary neutropenia could be an irAE.

Treating Rh-irAEs requires balancing the desire to decrease off-target inflammation while not negatively impacting the antitumor immune response. This means the treatment strategy ultimately depends on the clinical presentation, severity of irAEs, and cancer treatment goals [10]. Horvat et al. have reported that glucocorticoid monotherapy or in combination with antitumor necrosis factor *α* (TNF*α*) therapy does not affect overall survival or time to treatment failure in patients with melanoma treated by ipilimumab monotherapy [11]. Roberts et al. suggested that treatment of Rh-irAEs did not affect the antitumor response to immunotherapy [4]. In contrast, combination immunosuppressive treatment for irAE management (steroids with other medications) resulted in poor ICI treatment efficacy compared to prednisolone monotherapy in ipilimumab and nivolumab treatment for melanoma [12]. In addition, Bass et al. have shown that TNF*α* inhibitor use for Rh-irAE treatment shortened the time to cancer progression compared to methotrexate [13]. Mitchell et al. have also reported the possibility of decreased ICI efficacy by prolonged immunomodulation in patients receiving anti-programmed death-1 antibodies [5]. These conflicting results further highlight the need to balance irAE management and ICI efficacy.

However, management of Rh-irAEs is important to maintain the quality of life and daily activities of patients. Corticosteroid monotherapy is a general treatment for Rh-irAEs; however, its management can take a long time [1, 4]. In this case, the symptoms rapidly responded to 15 mg of prednisolone; however, it was difficult to reduce its dosage. Long-term systemic corticosteroid administration induces various adverse effects, such as gastrointestinal symptoms, insomnia, infection, loss of bone density, and blood sugar elevation. As the patient had already been administered certain medications before prednisolone administration, we only initiated trimethoprim-sulfamethoxazole from corticosteroid initiation to successfully prevent pneumocystis pneumonia. Disease-modifying antirheumatic drugs (DMARDs) are an additional effective treatment in cases for which prednisolone reduction is difficult [1, 5]. Iguratimod suppresses immunoglobulin production through its direct action on B lymphocytes as well as inflammatory cytokine production through its inhibitory action on nuclear factor-kappa B activation [14]. It appears to cause fewer adverse reactions than those of other immunosuppressive agents as it has a milder inhibitory action on lymphocyte proliferation [14]. Hara et al. have suggested the noninferiority of iguratimod monotherapy to salazosulfapyridine [15]. Nozaki et al. also reported that iguratimod monotherapy shows a high retention rate and good efficacy without an increased risk of serious adverse events [16]. As patients with cancer receiving chemotherapy often exhibit adverse effects or paraneoplastic symptoms, we chose this drug even though its impact on ICI efficacy was unclear, which resulted in symptom improvement in this case. Consequently, a clear-cut answer for the impact of immune-suppressive treatment on the efficacy of ICI treatment over longer evaluation periods is necessary.

Baseline predictors of Rh-irAEs and de novo inflammatory arthritis are melanoma, genitourinary cancer, combination ICI treatment, preexisting autoimmune disease, and recent glucocorticoid use [17]. We should carefully monitor patients with these risk factors for Rh-irAE development although this patient did not have the above factors. In addition, it is occasionally difficult to diagnose Rh-irAEs because certain patients consider the symptoms manageable and unproblematic. However, because erosive joint damage or changes progress rapidly in many cases, permanent motion limitation or chronic symptoms may occur in cases of delayed diagnosis [18]. Fortunately, the symptoms advanced relatively slowly from grade 1 to 2 over approximately 4 months, and the patient responded well to prednisolone in this case. However, the possibility of rapid symptom progression, the multisystem feature of irAEs, and appropriate immune-suppressive induction should be considered for adequate Rh-irAE management.

In conclusion, we reported a case of significantly delayed polyarthritis with active tenosynovitis after possible temporary hematological irAEs. These findings emphasize the need for careful monitoring of irAEs.

## Figures and Tables

**Table 1:** Laboratory data on day 645.

	Value on day 645	Normal range
IgA (mg/dL)	453	93–393
IgG (mg/dL)	2510	861–1747
IgM (mg/dL)	186	33–183
Complement C3 (mg/dL)	180	73–138
Complement C4 (mg/dL)	46	11–31
50% hemolytic complement activity (U/mL)	90.7	31.6–57.6
Anti-nuclear antibody (fold)	40.0	<40
SS-A antibody (U/mL)	>2200	<10
PR3-ANCA (U/mL)	15.0	<3.5
MMP-3 (ng/mL)	430.5	36.9–121.0
C-reactive protein (mg/dL)	3.08	0–0.14
MPO-ANCA (U/mL)	<0.3	<3.5
Rheumatoid factor (IU/mL)	<4.5	≤15
Uric acid (mg/dL)	5.7	3.7–7.8
Anti-citrullinated peptide antibody	Negative	Negative

## Data Availability

All data generated during this study are included in this article. Further enquiries can be directed to the corresponding author.
